# The complete mitochondrial genome sequence of *Liobagrus mediadiposalis* (Teleostei, Siluriformes, Amblycipitidae)

**DOI:** 10.1080/23802359.2017.1407702

**Published:** 2017-11-27

**Authors:** Chang Eon Park, Min-Chul Kim, Kgu-Hwan Kim, Hee Cheon Park, Jae-Ho Shin

**Affiliations:** a School of Applied Biosciences, Kyungpook National University, Daegu, Republic of Korea;; b Institute of Ornithology, Association of Ex-situ Conservation, Daegu, Republic of Korea;; c Department of Radiologic Technology, Daegu Health College, Daegu, Republic of Korea;; d Institute of Agricultural Science & Technology, Kyungpook National University, Daegu, Republic of Korea

**Keywords:** Amblycipitidae, Korean endemic species, *Liobagrus mediadiposalis*, south torrent catfish

## Abstract

The south torrent catfish *Liobagrus mediadiposalis* (Teleostei, Siluriformes, Amblycipitidae) is an endemic species of Korea. The complete mitochondrial genome sequence consisted of 16,534 base pairs (bp) encoding 13 protein-coding genes (PCGs), two ribosomal RNAs (rRNAs), 22 transfer RNAs (tRNAs) and two non-coding regions. The overall base composition of *L. mediadiposalis* was G + C: 44.8%, A + T: 55.2%, apparently with a slight AT bias. Phylogenetic analysis showed that *L. mediadiposalis* was closely related to *Liobagrus reinii.*

*Liobagrus mediadiposalis* (Teleostei, Siluriformes, Amblycipitidae) is called south torrent catfish endemic to Korea. Currently, 15 recognized species in the *Liobagrus* fishes are distributed in China, Taiwan, Japan and Korea (Zhao et al. 2004). Among them, five catfishes occur in Korea: *L. andersoni, L. hyeongsanensis, L. obesus, L. somjinensis* and *L. mediadiposalis* (Park and Kim 2010; Lee et al. 2014; Kim et al. 2015). Complete mitogenome sequences of *L. andersoni* (Lee et al. 2016) and *L. obesus* (Kartavtsev et al. 2007) were previously reported. Here we report the complete mitochondrial genome of *L. mediadiposalis* (GeneBank accession number: KR075136).

A whole body specimen of adult south torrent catfish is being kept in Institute of Ornithology, Daegu, Korea. The sample was obtained from the same institute. The mitochondrial DNA was amplified using the modified mitochondrial specific primer sets (Miya and Nishida 2000). The mitochondrial DNA was amplified into two overlapping segments by a long-range PCR method. DNA shotgun sequencing was operated by an Ion PGM™ system (Life Technologies, Gaithersburg, MD), and genome assembly was carried out using the CLC Genomics Workbench 7.5 program (CLC Bio, Aarhus, Denmark).

The complete mitogenome was 16,534 bp in size, consisting of 13 protein-coding genes (PCGs), two ribosomal RNAs (rRNAs) genes, 22 transfer RNAs (tRNAs) and two non-coding region (D-loop and origin of light strand replication). All the PCGs (*ND1*, *ND2*, *ATP8*, *ATP6*, *CO3*, *ND3*, *ND4L*, *ND4*, *ND5*, *ND6*, *CytB*) shared start codon ‘ATG’, except for CO1 (start codon ‘GTG’). Regarding the stop codons, five PCGs (*CO1*, *ATP8*, *ATP6*, *ND4L*, *ND5*) shared stop codon ‘TAA’, two PCGs (*ND1*, *ND6*) shared stop codon ‘TAG’ and six PCGs (*ND2*, *CO2*, *CO3*, *ND3*, *ND4*, *CytB*) shared the incomplete stop codon ‘T’. The two rRNA genes were 12S rRNA (956 bases) and 16S rRNA (1670 bases). The D-loop (908 bases) was located between tRNA-Glu and tRNA-Phe. The origin of light strand replication (31 bases) was located between tRNA-Asn and tRNA-Cys. The overall base composition of *L. mediadiposalis* was G: 16.1%, C: 28.7%, A: 30.1%, T: 25.1%, apparently with a slight AT bias (G + C: 44.8%, A + T: 55.2%).

We compared the results from the present study to previous mitogenome research on the genus *Liobagrus* (Kartavtsev et al. 2007; Jia et al. 2013a, 2013b, 2013c; Huang et al. 2017). Phylogenetic analysis based on the mitogenome sequences using the maximum likelihood approach showed that the *L. mediadiposalis* was closely related to *Liobagrus reinii* (Figure 1). Thus, the *L. mediadiposalis* mitogenome sequence can contribute to phylogenetic knowledge of the genus *Liobagrus* and expand the basis of Korean endemic species.

**Figure 1.**
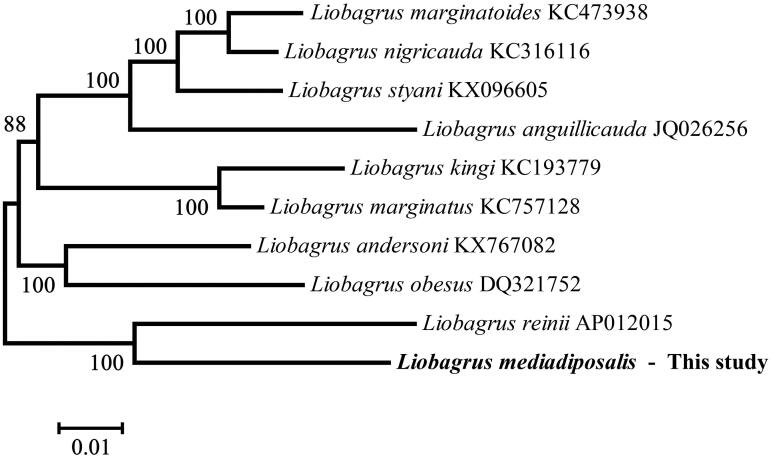
Phylogenetic tree based on 10 whole mitogenomes constructed using maximum likelihood approach. The number in the phylogenetic tree is bootstrap probability value and presented in the above branches. The GenBank accession numbers are indicated after the scientific name.
